# Regulation of Apoptosis and Oxidative Stress by Oral Boswellia Serrata Gum Resin Extract in a Rat Model of Endometriosis

**DOI:** 10.3390/ijms232315348

**Published:** 2022-12-05

**Authors:** Ramona D’Amico, Daniela Impellizzeri, Marika Cordaro, Rosalba Siracusa, Livia Interdonato, Rosalia Crupi, Enrico Gugliandolo, Francesco Macrì, Davide Di Paola, Alessio Filippo Peritore, Roberta Fusco, Salvatore Cuzzocrea, Rosanna Di Paola

**Affiliations:** 1 Department of Chemical, Biological, Pharmaceutical and Environmental Sciences, University of Messina, Viale Ferdinando Stagno D’Alcontres, n 31, 98166 Messina, Italy; 2 Department of Biomedical, Dental and Morphological and Functional Imaging, University of Messina, Via Consolare Valeria, 98125 Messina, Italy; 3 Department of Veterinary Sciences, University of Messina, Viale Annunzita, 98168 Messina, Italy

**Keywords:** endometriosis, oxidative stress, apoptosis

## Abstract

Endometriosis (EMS) is a gynecological disease characterized by inflammation, oxidative stress, and apoptosis dysregulation. This study aims to evaluate the effect of Boswellia serrata gum resin extract (BS) on the endometriotic lesions in a rat model of endometriosis. We divided female rats into three groups, including Sham, EMS, EMS + BS. In the EMS and EMS + BS groups, pathology was induced and after 7 days by the abdominal high-frequency ultrasound (hfUS) analysis the presence of the endometriotic lesions was confirmed. Subsequently, the EMS + BS group was administered with BS (100 mg/Kg) daily for another 7 days. At the end of the experiment, the hfUS analysis was repeated and the animals were sacrificed to evaluate the size and histoarchitecture of the endometriotic implants. Pelvic ultrasound showed increased size of the endometriotic lesions in the Endo group, while BS administration reduced the lesion size. The macroscopic analysis confirmed the reduced area and volume of the endometriotic lesions of the EMS + BS group. The histological analysis showed reduced characteristic of ectopic stroma and glands in the animals treated with BS. Western blot analyses were conducted to evaluate the nuclear factor erythroid 2-related factor 2 (Nrf2) pathway. BS increases the expression of Nfr2 in the nucleus and the expression of its downstream antioxidant proteins NQO-1 and HO-1. Moreover, it reduced lipid peroxidation and increased glutathione (GSH) levels, and glutathione peroxidase (GPx) and superoxide dismutase (SOD) activities. BS administration also restored the impaired apoptotic pathway in the lesions by reducing Bcl-2 expression and increasing Bax and cleaved caspase 9 levels. The BS apoptotic effect was also confirmed by the cleavage of PARP, another specific marker of apoptosis, and by the TUNEL assay. Our results show that BS administration resulted in an effective and coordinated suppression of Endo owing to its antioxidant and antiapoptotic activities.

## 1. Introduction

Endometriosis (EMS) is a common pathology characterized by the growth of the endometriotic tissues outside of the uterine cavity [1]. The main symptoms of the pathology are infertility, chronic pelvic pain, menstrual irregularity, and dyspareunia. All these features result in a reduced quality of life of patients [2]. How the pathology starts and develops is still under investigation. The most accepted theory is the Sampson’s hypothesis in which the retrograde menstruation phenomenon has a key role. According to Sampson, during menstruation, fragments of endometriotic tissue migrate outside the cavity and reach the peritoneum. Here the tissue adheres to the walls of the peritoneal cavity and develops in endometriotic lesions. Histologically these endometriotic lesions are characterized by an external tissue that encapsulates the epithelial cells and the stroma. From the molecular point of view the ectopic endometrium is characterized by dysregulated homeostasis of the anti-inflammatory, anti-oxidative, and antiapoptotic pathways. Retrograde menstruation permits transport of pro-oxidant mediators, such as heme, apoptotic endometriotic cells, and iron, which are well-known activators of oxidative stress, into the peritoneum of women with EMS [3]. 

Reactive oxygen species (ROS) are responsible for the promotion of the growth of endometriotic stromal cells inducing hyperproliferation and reducing apoptosis [4]. Redox-sensitive nuclear factor erythroid-derived 2-like 2 (Nrf2) is responsible for the control of the transcription of endogenous antioxidant enzymes and protects against oxidative injury which is generated by inflammation and oxidative stress, thereby promoting the development of EMS [5]. It has been demonstrated that Nrf2-mediated modulation of cell death has a key role in this pathology. Furthermore, endometriotic lesions are characterized by impaired apoptotic pathway [6]. Apoptosis has a key role in maintaining tissue homeostasis by eliminating dysfunctional and excess cells. Bcl-2 family proteins are the main mediators involved in the pathway. In particular, Bcl-2 and Bax proteins participate in the process promoting and preventing apoptosis [7]. The Bax protein promotes a cascade effect releasing cytochrome c from mitochondria and inducing cell death. On the contrary, Bcl-2 blocks Bax activity and inhibits the activation of apoptotic machinery [8]. Several agents are used for the treatment and management of EMS.

In recent years, botanical products and medicinal herbs have become popular for gynecological disorders [9,10,11,12] including EMS [13,14]. Evidence of the efficacy of natural substances in EMS-associated symptoms has been described in the literature [15].

Boswellia serrata belongs to the family Burseraceae and has been widely used for management of inflammatory diseases [16,17]. It includes several compounds, chemically characterized by pentacyclic triterpenoid structures [18], in the gum resin that is responsible for pharmacological effects [19,20]. In particular, the Boswellia serrata gum resin extract (BS) showed important anti-oxidant and anti-inflammatory activities in many in vivo experimental models including myocardial I/R injury [21], bowel disease [22], and pulmonary fibrosis [23]. Indeed BS showed neuroprotective activities on H2O2-induced injury in vitro [24] and cerebral ischemic damage in mice by activation of the Nrf2 pathway [25]. BS also displayed efficacy on several tumor cell lines [26,27,28,29,30] showing anticancer activity by inducing apoptosis and preventing cell proliferation. 

Starting from these findings, we aimed to evaluate the effect of oral BD administration in a rat model of EMS, in particular, investigating its effects on oxidative stress-induced impaired apoptosis during the pathology.

## 2. Results

### 2.1. Monitoring of Endometriotic Lesions Development

**Figure 1:**
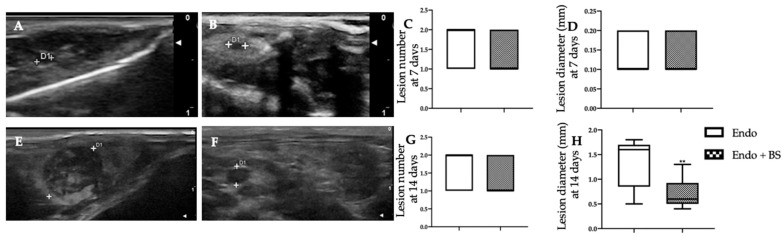
Analysis of endometriotic lesions development: high-frequency ultrasound analysis (hfUS) analysis at 7 days from the surgery: EMS (**A**), EMS + BS (**B**), lesion number (**C**), lesion diameter (**D**); hfUS analysis at 14 days from the surgery: EMS (**E**), EMS + BS (**F**), lesion number (**G**), lesion diameter (**H**). For the analyses, *n* = 5 animals from each group were employed. A *p*-value of less than 0.05 was considered significant. ** *p* < 0.01 vs. control.

Endometriotic lesions were detected seven days from the induction in the inner surface of the peritoneal cavity in both groups (Figure 1A,B) and no difference were detected in lesions number (Figure 1C) and diameter (Figure 1D). Rats were divided into two groups, untreated (EMS) and orally administered BS (EMS + BS) from the seventh day until the fourteenth day. Fourteen days from the induction pelvic ultrasound showed the same number of endometriotic lesions in both groups (Figure 1G), but in the EMS + BS group the lesion diameter was significantly reduced (Figure 1F,H), as compared to the EMS group (Figure 1E,H, ** *p* < 0.01 vs. control).

### 2.2. Effect of Oral BS Administration on Macroscopic and Histological Analysis

**Figure 2:**
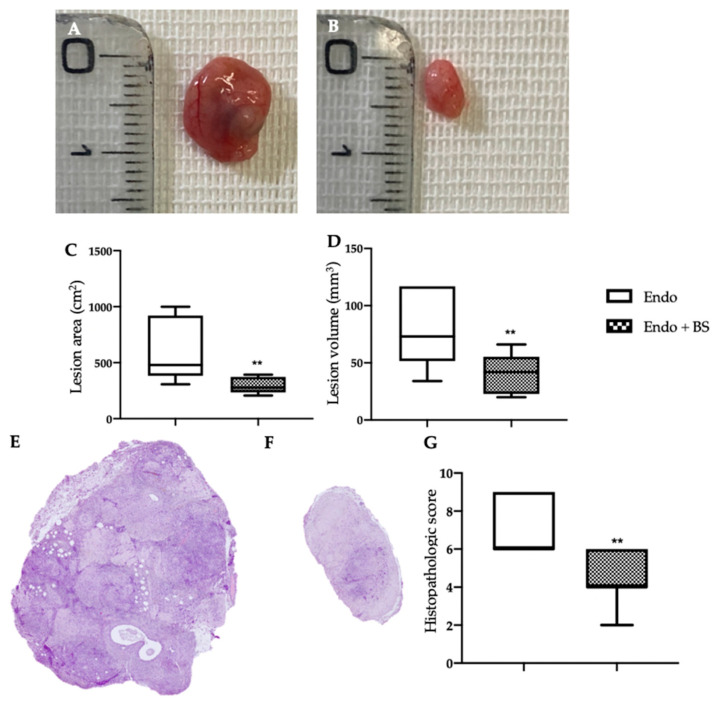
Analysis of the effect of oral BS administration on lesions size and histology: Macroscopic analysis: EMS (**A**), EMS + BS (**B**); lesion area (**C**); lesion volume (**D**); histological analysis: EMS (**E**), EMS + BS (**F**), histopathologic score (**G**). For the analyses, *n* = 5 animals from each group were employed. A *p*-value of less than 0.05 was considered significant. ** *p* < 0.01 vs. control.

The macroscopic endometrioma analysis (Figure 2A,B) was in line with the hfUS examination. Lesions from the EMS group had major area (Figure 2C, ** *p* < 0.01 vs. control) and volume (Figure 2D, ** *p* < 0.01 vs. control), as compared to the one harvested from the EMS + BS group. The histological exam revealed that oral BS administration also modified lesions morphology (Figure 2G, ** *p* < 0.01 vs. control). Endometriotic lesions from EMS group showed characteristic stroma and glands (Figure 2E), while the oral BS administration (Figure 2F) significantly reduced the histopathological score (Figure 2G). 

### 2.3. Effect of Oral BS Administration on the Oxidative Stress

**Figure 3:**
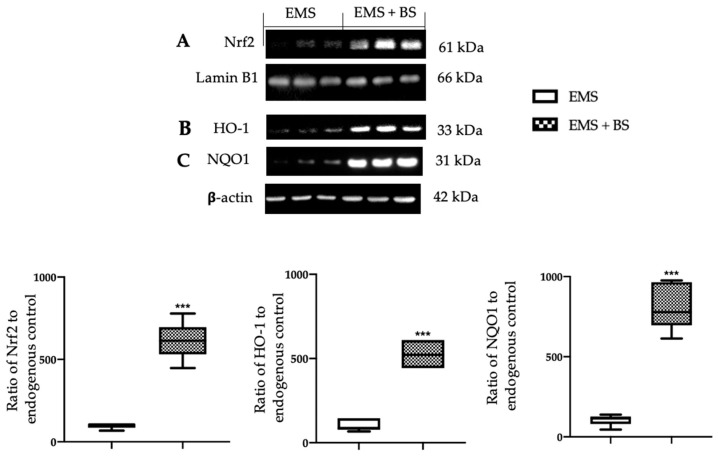
Analysis of the effect of oral BS administration on Nrf2 pathway: Western blot analysis of: Nrf2 (**A**), HO-1 (**B**), NQO-1 (**C**) expression; for the analyses, *n* = 5 animals from each group were employed. A *p*-value of less than 0.05 was considered significant. *** *p* < 0.001 vs. control.

**Figure 4:**
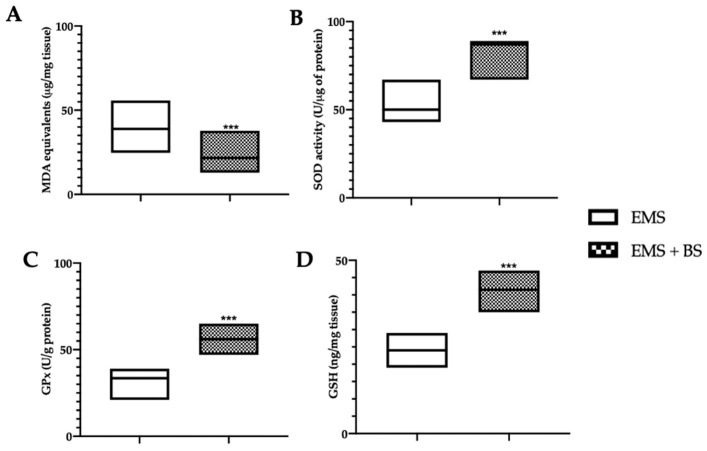
Analysis of the effect of oral BS administration on oxidative stress: MDA levels (**A**), SOD activity (**B**), GPx activity (**C**), GSH levels (**D**). For the analyses, *n* = 5 animals from each group were employed. A *p*-value of less than 0.05 was considered significant. *** *p* < 0.001 vs. control.

Western blot analysis showed a significant increase in the expression of nuclear Nrf2 (Figure 3A, *** *p* < 0.01 vs. control) and cytosolic HO-1 (Figure 3B, *** *p* < 0.01 vs. control) and NQO-1 (Figure 3C, *** *p* < 0.01 vs. control) levels in the EMS + BS group, as compared to the EMS group. Moreover, oral BS administration significantly reduced malondialdehyde (MDA) levels (Figure 4A, *** *p* < 0.01 vs. control), increased superoxide dismutase (SOD) (Figure 4B, *** *p* < 0.01 vs. control) and glutathione peroxidase (GPx) (Figure 4C, *** *p* < 0.01 vs. control) activities and glutathione (GSH) (Figure 4D, *** *p* < 0.01 vs. control) levels.

### 2.4. Effect of Oral BS Administration on the Apoptosis Pathway

**Figure 5:**
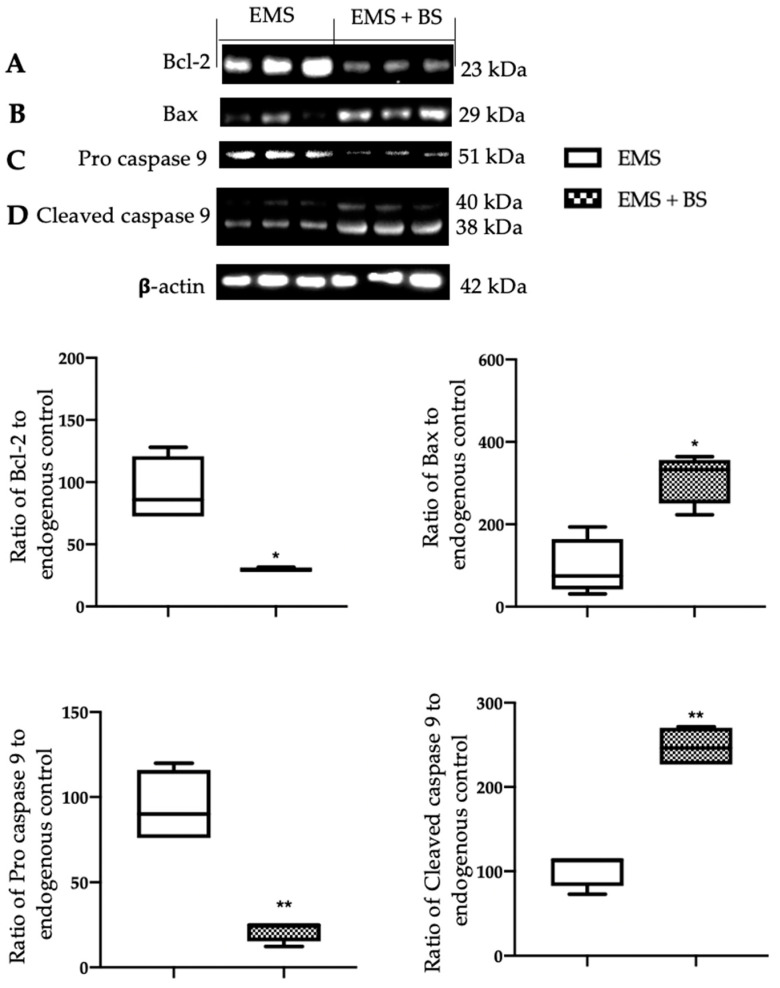
Analysis of the effect of oral BS administration on apoptosis: Western blot analysis of: Bcl-2 (**A**), Bax (**B**), Pro caspase 9 (**C**), cleaved caspase 9 (**D**). For the analyses, *n* = 5 animals from each group were employed. A *p*-value of less than 0.05 was considered significant. * *p* < 0.05 vs. control, ** *p* < 0.01 vs. control.

**Figure 6:**
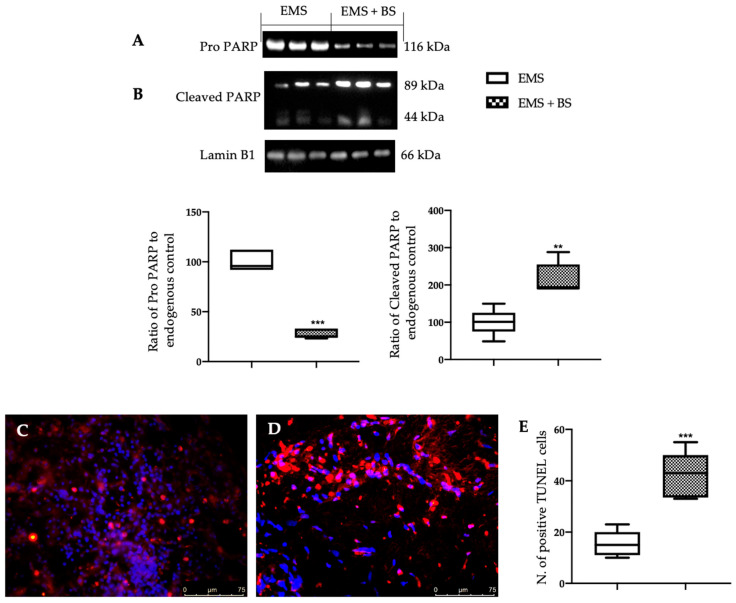
Analysis of the effect of oral BS administration on apoptosis: Western blot analysis of: Pro PARP (**A**), cleaved PARP (**B**) expression; TUNEL staining (magnification 40×): EMS (**C**), EMS + BS (**D**), number of TUNEL positive cells (**E**). For the analyses, *n* = 5 animals from each group were employed. A *p*-value of less than 0.05 was considered significant. ** *p* < 0.01 vs. control, *** *p* < 0.001 vs. control.

Western blot analysis showed that samples harvested from the EMS + BS group had a significant reduction in Bcl-2 (Figure 5A, * *p* < 0.01 vs. control) and Pro caspase 9 (Figure 5C, ** *p* < 0.01 vs. control) levels, as compared to EMS group. Levels of Bax (Figure 5B, * *p* < 0.01 vs. control) and cleaved caspase 9 (Figure 5D, ** *p* < 0.01 vs. control) were significantly increased in the EMS + BS samples, as compared to EMS. Additionally, Pro PARP expression was significantly reduced in EMS + BS group (Figure 6A, *** *p* < 0.01 vs. control), while cleaved PARP increased (Figure 6B, ** *p* < 0.01 vs. control), as compared to the EMS group. TUNEL staining confirmed these data. A significant increased number of TUNEL positive cells were detected in the EMS + BS group (Figure 6D,E, *** *p* < 0.01 vs. control), as compared to the EMS group (Figure 6C,E, *** *p* < 0.01 vs. control). 

## 3. Discussion

The molecular mechanism underlying the pathophysiology of the EMS is controversial, as the therapeutic treatments. Several studies proposed the antioxidants as beneficial tools for EMS [31,32]. In this paper, BS, as an antioxidant agent and apoptosis inducer were used in an animal model of EMS. The pathology was induced and monitored by hfUS analysis. The macroscopic endometrioma analysis were in line with the hfUS examination. Additionally, BS administration modified endometriotic lesions histology, reducing glands and stroma tissue. Oxidative stress plays a key role in the establishment and development of the endometriosis [33]. Endometriotic cells showed an unbalanced oxidative/antioxidative, with an alteration of the ROS detoxification pathways [34]. The activity of Nrf2 is decisive to maintain intracellular oxidative stress status [35]. In fact, the Nrf2 transcription factor and its downstream proteins HO-1 and NQO1 were elected as one of the main pathways involved in the disease [36]. Nrf2 activity is normally restricted by its negative regulator Keap1. Increased oxidative stress induces Keap1 degradation, which in turn leads to Nrf2 translocation into the nucleus. Once translocated Nrf2 binds to the anti-oxidant response element (ARE) and promotes the transcription of its downstream antioxidant effectors [37]. In the endometriotic lesions the endogenous antioxidant systems are compromised and the oxidative/antioxidative equilibrium is unbalance. BS administration, well in line with the previous evidences [25], increased the Nrf2 nuclear levels and the expression of the downstream proteins HO-1 and NQO1. Additionally, it reduced lipid peroxidation and increased SOD and GPx activities, restoring the reduced GSH levels. Thus, BS restored the oxidative imbalance activating the endogenous antioxidant defense mechanisms. 

The increased oxidative stress in the endometriotic lesions also impaired the apoptotic pathway [6]. It has been demonstrated that Nrf2-mediated modulation of apoptotic cell death has a key role in cell survival and drug resistance. In particular, impaired Nrf2 pathway results in increased expression of antiapoptotic protein Bcl-2 and reduced Bax levels, cytochrome c release from mitochondria, modulation of caspases, and DNA fragmentation [38]. Defective control of the programmed cell death has been shown to have an important role in the establishment of several disease including endometriosis [39]. Bcl-2 family proteins control the apoptotic mitochondria-dependent pathway. Several papers reported that the increase in the anti-apoptotic protein Bcl-2 hasten the progression of endometriosis [40]. The up-regulated Bcl-2 is accompanied with reduced Bax levels and caspases cleavage [41]. Caspases are endoproteases that have a key role in controlling apoptosis. In particular, it has been reported that in the endometriotic lesions the activation of caspase 9 is impaired [42]. As already confirmed by other studies [26,27,28,29,30], our results showed that BS caused a decrease in Bcl-2 expression and an increase in Bax levels and caspase 9 cleavage. The BS apoptotic effect was also confirmed by the cleavage of PARP, another specific marker of apoptosis, and by the TUNEL assay. PARP is a nuclear protein that promotes the transfer of ADP-ribose polymers onto itself and other nuclear enzymes in response to DNA strand breaks. TUNEL assays detect apoptotic cells by the terminal deoxynucleotidyl transferase (TdT)-mediated addition of labeled (X) deoxyuridine triphosphate nucleotides (X-dUTPs) to the 3′-OH end of DNA strand breaks. During apoptosis, cleavage of PARP-1 in fragments and TUNEL positive cells are a useful hallmark of apoptosis or cell death. Our experimental evidence showed that BS administration was able to restore the impaired apoptosis signaling. TUNEL staining confirmed these data.

There are several limitations in the current study. A normal rat’s uterine tissue transplant into another rat’s abdominal cavity was employed as the endometriosis model in this study. It was a poor representation of the ethology of endometriosis in humans. Indeed, the data were gathered using a synthetic model (no spontaneous lesions, no rat menstruation, no human lesion transplants). Rat models, on the other hand, have a long history of being employed extensively in endometriosis research and have also been approved as a model that captures the dynamics of the condition. It would be interesting to investigate the lesions for a longer duration in subsequent studies.

## 4. Materials and Methods

### 4.1. Animals

Sprague–Dawley rats (Envigo, Milan, Italy) were employed. The University of Messina Review Board for animal care (OPBA) approved the study. All animal experiments agree with the new Italian regulations (D.Lgs 2014/26), EU regulations (EU Directive 2010/63).

### 4.2. Model Induction

Rats were divided into two groups, donor or recipient. Donor animals were administered intraperitoneally with 10 IU pregnant mare’s serum gonadotropin (PMSG) to induce comparable estrogen levels among them. Donors were euthanized 41 h after the injection. The uterus was removed through a midline incision and washed in PBS before extrauterine tissue, including ovary and oviduct, was removed under a dissecting microscope. A longitudinal incision was made from one horn to the other. Tissue was transferred to a 1.5 mL centrifuge tube containing fresh PBS and minced with dissecting scissors. Minced tissue from all donors was pooled and the volume was adjusted to the equivalent of one uterus/500 uL of PBS. Recipient rats were injected intraperitoneally with the equivalent of tissue from one uterus in 500 uL of PBS (1:1 donor/recipient ratio) along the midventral line using a 18-gauge needle [43]. The disease was allowed to establish for seven days. 

### 4.3. Experimental Groups

Seven days after the induction, recipient rats were assigned to the following groups (*n* = 20 for each group):

- EMS group: animals were subjected to the above described induction and vehicle (2% gum acacia was orally administered on the seventh day until the fourteenth day);
- EMS + BS group: animals were subjected to the above described induction and BS (100 mg/Kg) was orally administered on the seventh day until the fourteenth day;
- Sham group: animals were subjected to the above described induction but they were intraperitoneally injected with 500 μL of PBS along the midventral line instead of endometriotic tissue.

BS dosage was based on previous studies [44].

The powder of BS was purchased from a company (Fontana standardized natural active principles, lot. S2111560, Canosa di Puglia, BT Italy).

**Figure 7:**
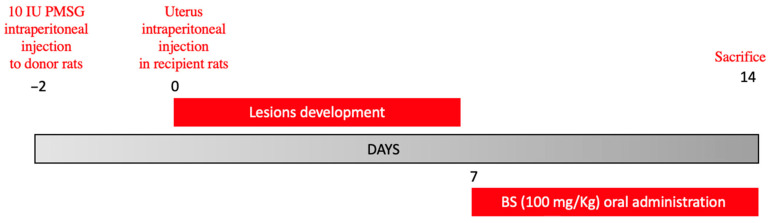
Study design figure.

Fourteen days from the endometriosis induction, animals were sacrificed and the endometriotic lesions were collected for the histological and molecular analyses (Figure 7). 

### 4.4. Abdominal High-Frequency Ultrasound

Pelvic ultrasound was performed to monitor the development of the endometriotic lesions at seven and fourteen days from the implant. The analysis included the anterior and posterior pelvic areas to reach the lesions in both locations. The hair in the ventral abdomen was clipped from 1 cm cranial to the xyphoid cartilage to the caudal-most part of the pubis. Ultrasonographic exams were performed by the Esaote MYLAB OMEGA VET (Esaote Italia, Milan, Italy) on anesthetized rats (2% isoflurane) positioned in dorsal recumbency. Abdominal B-mode was performed with a high frequency linear array (4–15 MHz) transducer. Longitudinal and transverse scanning planes were employed for evaluation of different abdominal structures [45]. All analyses were performed as double blinded. 

### 4.5. Biochemical Analysis

Lipid peroxidation was evaluated by the TBARS test, reading the MDA levels at 535 nm [46]. SOD activity was evaluated as already described [47] and expressed as U/g protein [48]. GSH levels were determined using a microplate reader at 412 nm [49]. GPx activity was evaluated as already described [50] and expressed as U/g protein [51].

### 4.6. Histological Examination

Endometriotic lesions were fixed in buffered formaldehyde solution, dehydrated and embedded in Paraplast [52,53]. Tissue slides were stained with H&E and evaluated using a Leica DM6 microscope (Leica Microsystems SpA, Milan, Italy) [54]. Histopathologic scores were evaluated with the formula P (persistence of epithelial cells in the explants) × I (intensity of glands) as already described [55]: P: 3 = well-preserved epithelial layer, 2 = moderately preserved epithelium with leukocyte infiltrating, 1 = poorly preserved epithelium (occasional epithelial cells only), and 0 = no epithelium; I: from 0 (no glands) to 3 (abundant glands). Lesion volume was calculated according to the formula: V = (length × width2) × 0.5.

### 4.7. Western Blot Analysis

Lesion samples were homogenized and Western blots were performed as already described [56]. Specific primary antibody anti-Bcl-2 (Santa Cruz Biotechnology (Santa Cruz, CA, USA), sc-7382, 1:2000), anti-Bax (Santa Cruz Biotechnology, sc-7480, 1:2000), anti-Pro caspase 9 (Santa Cruz Biotechnology, sc-56073, 1:1000), anti-cleaved caspase 9 (Cell Signalling (Danvers, MA, USA) 9509, 1:1000), anti-Pro PARP (Santa Cruz Biotechnology, sc-8007, 1:1000), anti-cleaved PARP (Cell Signalling 5625, 1:1000), anti-Nrf2 (Santa Cruz Biotechnology, sc-365949, 1:1000), anti-HO-1 (Santa Cruz Biotechnology, sc-390991, 1:2000), anti-NQO-1 (Santa Cruz Biotechnology, sc-32793, 1:2000), were mixed in 5% *w*/*v* nonfat dried milk solution and incubated overnight. Afterward, blots were incubated with peroxidase-conjugated anti-mouse IgG secondary antibody or peroxidase-conjugated ant-irabbit IgG (Jackson Immuno Research, Milan, Italy) for 1 h at room temperature [57]. Membranes were also blotted with the antibody against β-actin or lamin b1 [58]. Signals were detected with enhanced chemiluminescence detection system reagent (Super-SignalWest Pico Chemiluminescent Substrate) [59]. The relative expression of the protein bands was quantified by densitometry with Bio-Rad ChemiDoc XRS software (Bio-Rad, Milan, Italy) [60] and standardized to β-actin or lamin B1 levels. Images of blot signals were imported to analysis software (v2003, Image Quant TL) [61].

### 4.8. Terminal Deoxynucleotidyl Nick-End Labeling (TUNEL) Assay

Apoptosis was analyzed by a TUNEL assay using an in situ cell death detection kit (Roche 11684795910) [62]. 

### 4.9. Statistical Analysis

All values are expressed as mean ± standard error of the mean of N observations. The results were analyzed by *t*-test when comparing two groups while we used the one-way ANOVA followed by a Bonferroni post hoc test for multiple comparisons. A *p*-value of less than 0.05 was considered significant. * *p* < 0.05 vs. control, ** *p* < 0.01 vs. control, *** *p* < 0.001 vs. control.

## 5. Conclusions

BS is generally well tolerated, although it can have some side-effects [63,64,65]. Overall, this study was conducted to evaluate the effect of oral administration of BS in a rat model of endometriosis.

The results showed that BS works on the Nrf2 pathway to apply its antioxidant effects and that it induces early apoptosis acting on Bcl-2 and Bax expression and cleaving caspase 9 and PARP. Importantly, our results showed a response to the test material (Boswellia Serrata gum resin extract), allowing future studies to continue to develop this model.

## Data Availability

The data presented in this study are available on request from the corresponding author.
